# Can biochar and late-season nitrogen fertilization overcome the sulfur-containing amino acid deficiency and improve oil stability in soybean seeds?

**DOI:** 10.3389/fpls.2026.1849670

**Published:** 2026-05-19

**Authors:** Deepak Khatri, Sudip Poudel, Lalit Pun Magar, Manish Pandit, Anuj Chiluwal

**Affiliations:** College of Agriculture, Health, and Natural Resources, Kentucky State University, Frankfort, KY, United States

**Keywords:** amino acids, biochar, fatty acids, nitrogen fertilization, seed quality, soybean

## Abstract

Soybean meal quality for monogastric animals is limited by low sulfur-containing amino acids, especially methionine and cysteine, while oil oxidative stability is restricted by the high polyunsaturated fatty acid content. This two-year field study (2023-2024) evaluated the effects of biochar (0 and 12 t ha^-1^), cultivars differing in maturity group (MG2 and MG4), and late-season nitrogen (0, 40, 80, and 120 kg N ha^-1^ at R5) on ten essential amino acids and five major fatty acids in soybean seeds using a split-split plot randomized complete block design. Late-season nitrogen at 80 kg ha^-1^ (N80) and 120 kg ha^-1^ (N120) increased the concentration of seven out of ten essential amino acids, including threonine and lysine, but did not improve the levels of methionine, cysteine, or tryptophan. Amino acid concentrations varied by year, and their responses were most pronounced in the warmer 2024 season. The growing season also affected seven amino acids between cultivars, with the MG2 cultivar accumulating 3.4-5.9% higher concentrations than the MG4 cultivar under 2024 conditions. For fatty acids, cultivar and year interaction was the dominant factor shaping seed oil quality. MG2 cultivar had a higher (55.3%) oleic to polyunsaturated fatty acids ratio [Oleic/(Linoleic + Linolenic)] compared to MG4 cultivar in 2023, while nitrogen (N80 and N120) only changed oleic acid (p = 0.048) without improving the overall O/(L+Ln) ratio. Biochar at 12 t ha^-1^ had no significant influence on both amino acid and fatty acid concentrations. These results highlight that biochar and nitrogen fertilization may not address the sulfur-containing amino acid deficiency or oil stability, indicating that alternative strategies will be necessary to improve soybean seed quality.

## 1 Introduction

Soybean (*Glycine max* L.) is one of the most important nutritional crops in the United States and was the second most grown crop by area of production after corn in 2025, with a production area of 80.437 million acres (USDA, 2025). The U.S. is the second-largest producer of soybeans after Brazil, producing approximately 116 million metric tons in the 2025/26 marketing year, and together with Brazil and Argentina, these three countries account for approximately 80% of the global soybean production (USDA-FAS, 2025). This production represents a significant component of the U.S farm economy. Of the total U.S. soybean production, approximately 43% was exported and 56% was crushed domestically for meal and oil in the 2024/25 marketing year (USDA-FAS, 2025). This rising global demand for soybean, both for protein-rich feed and vegetable oil, has made improving soybean seed quality through agronomic management a critical research priority.

Soybeans are valued for their nutrient composition and their seed comprises approximately 40% protein, 20% oil (fat), along with about 30-35% carbohydrates and 5 - 8% ash/fiber on a dry weight basis (Hou et al., 2009; Medic et al., 2014; Anwar et al., 2016). Protein and fat are the main components of soybean seeds, making up about 60% of the seed’s dry matter (Szostak et al., 2020). The high protein concentration and a well-balanced amino acid profile make soybean meal a key component of animal feed. The lower cost, balanced amino acids, and high digestibility and palatability establish soybean meal as the primary protein source for livestock and poultry (Park et al., 2002; Krishnan et al., 2005). In addition to the protein quantity in soybean meal, the protein quality is also important for poultry and livestock (Medic et al., 2014), as it directly influences their growth and production performance. A high protein concentration in soybean meals does not necessarily indicate high quality, as the relative abundance of amino acids is equally crucial (Medic et al., 2014). The balance of amino acids in soybean protein affects the quality and nutritional value of soybean meal (Thakur and Hurburgh, 2007). Although soybeans have a highly digestible amino acid profile, they are relatively low in sulfur-containing essential amino acids (Thakur and Hurburgh, 2007; Medic et al., 2014). Methionine is the most limiting amino acid, followed by cysteine, and to a lesser extent, threonine and lysine (Farkhoy et al., 2012; Warrington et al., 2015). Monogastric animals, such as poultry and pigs, are unable to synthesize these amino acids and must obtain them through supplements, which increases production costs (Medic et al., 2014; Warrington et al., 2015; Patil et al., 2017). Sometimes, supplemental methionine may lead to the formation of unwanted volatile sulfides (Warrington et al., 2015) and an excess of non-limiting amino acids (Medic et al., 2014) which further increases the cost of production. Consequently, it is crucial to develop high-yielding and protein-rich cultivars with elevated levels of these essential amino acids.

Similarly, the fatty acid composition of soybean oil has garnered significant attention because of the increasing demand for vegetable oil and health-related concerns (Clemente and Cahoon, 2009). Soybean oil is composed of five major fatty acids that occupied nearly 99% of the total fatty acids in the oil (Clemente and Cahoon, 2009; Anwar et al., 2016; Liu et al., 2020). Fully developed seeds upon maturation contain 15% saturated fatty acids, 23% monosaturated fatty acids, and 62% polyunsaturated fatty acids (Li et al., 2023). Palmitic acids (10-12%) and stearic acids (2.2-7.2%) are the major saturated fatty acids while oleic (24%), linoleic (54%), and linolenic (8%) are the predominant unsaturated fatty acids (Clemente and Cahoon, 2009; Bellaloui et al., 2012; Wilson, 2016; Liu et al., 2020). The proportion of these major fatty acids determines the soybean oil’s nutritional and industrial qualities (Tamagno et al., 2020). Among these, linoleic and linolenic acid are essential fatty acids for animals and humans and contribute to health benefits (Mensink et al., 2003), but they contain multiple double bonds which lead to a relatively low oxidative stability and become rancid upon exposure to heat, air or light that reduces the shelf-life in soybean oil (Clemente and Cahoon, 2009; Liu et al., 2020). On the other hand, oleic acid is a monounsaturated acid, which is more resistant to oxidation and has a longer shelf life. For this reason, breeding efforts are focused on improving soybean oxidative stability by increasing the oleic and saturated fatty acids like stearic (Clemente and Cahoon, 2009; Bellaloui et al., 2015). Thus, the fatty acid composition is a critical factor for consumer demand and end use of soybean oil.

Seed composition is mostly determined by genetics; however, environmental factors and effective agronomic practices, such as fertilizer application, also influence the seed composition (Rotundo and Westgate, 2009; Bellaloui et al., 2015, Bellaloui et al., 2020). Genetic differences among maturity groups significantly influence both protein and oil concentrations and their constituent amino acids and fatty acids profiles (Mourtzinis et al., 2017; Lee et al., 2019). A genome-wide association study across maturity groups I–IV identified multiple genomic regions associated with essential amino acid content, including methionine and cysteine (Lee et al., 2019). Similarly, significant genotypic variation has been documented for oleic, linoleic, and linolenic acid concentrations (Dar et al., 2017). While genetic improvement remains a long-term strategy for enhancing seed composition, agronomic interventions such as soil amendments and nutrient management offer more immediate and accessible approaches for producers. Biochar is a carbon-rich substance produced by burning organic biomass in an environment with low or no oxygen (Brassard et al., 2019; Semida et al., 2019; Diatta et al., 2020). It is a promising soil amendment that enhances soil properties in various ways due to characteristics like soil porosity, water retention capacity, carbon content, bulk density, soil pH, and cation exchange capacity (Murtaza et al., 2023), which aid in nutrient retention and boost the microbiome in the soil (Kabir et al., 2023). Biochar can indirectly impact soybean seed composition through its influence on soil fertility. It may enhance soybean plants to synthesize more protein by improving water, nitrogen, and micronutrient retention. Although studies of the impact of biochar on amino acids and fatty acids are emerging, some recently published articles suggest that biochar could alter the seed composition. A study by Arabi et al. (2018) found an increase in oil concentration while a decrease in protein when biochar application increased from 0 to 8 t ha^-1^. Similar results were also reported in another study where oil content was increased with no impact on protein (Wang et al., 2023). A study conducted in a controlled pot experiment found that soybean shoot tissue grown in biochar-amended soil showed higher levels of palmitic, stearic, and oleic acids compared to the control (Waqas et al., 2017). They also reported significantly higher levels of linolenic and linoleic acids in shoot tissue when biochar was combined with a beneficial endophytic fungus. The same study also observed notable increments in some of the essential amino acids, such as isoleucine, phenylalanine, methionine, and cysteine, when biochar was applied with fungus compared to the control. Similar alternations were recorded in the seed of lupine and winter rye with the application of biochar (Wiedner et al., 2019). While there are very limited direct investigations into soybean seed composition with biochar amendment, these results suggest that biochar application could potentially affect soybean seed composition.

Nitrogen is a major fundamental nutrient for plant growth and is a key component of amino acids (Zayed et al., 2023). Although soybeans meet a large portion of their nitrogen requirements through biological nitrogen fixation (48-93%), they are highly dependent on environmental conditions and cultivar yield potential (Salvagiotti et al., 2008; Cafaro La Menza et al., 2017; Tamagno and Ciampitti, 2017; Chiluwal et al., 2021). These studies suggest that under stressful environmental conditions and with high-yielding cultivars, biological nitrogen fixation alone cannot meet the demand. Thus, nitrogen supplementation in response to varying environments and modern cultivars is common practice. It was found that nitrogen application improved the plant during salinity stress in maize, tomato, wheat (Zayed et al., 2023). The effects of nitrogen application on soybean protein and oil content are documented in numerous studies (Wesley et al., 1998; Salvagiotti et al., 2009; Cafaro La Menza et al., 2017; de Borja Reis et al., 2020; Chiluwal et al., 2021; Głowacka et al., 2023), but the studies on amino acid and fatty acid profiles are very limited. One study found that nitrogen fertilization at 20 kg ha_-1_ significantly increased the majority of amino acids in the top dressing (Lee et al., 2024), and another study documented a decrease in sulfur-containing amino acids with the application of nitrogen (Krishnan et al., 2005). The fatty acid composition was not significantly influenced by the level of nitrogen application (Szostak et al., 2020). The application of sulfur along with nitrogen was found to increase the concentration of soybean protein and oleic acid, while decreasing the concentrations of oil and linolenic acid under irrigated conditions compared to the control group (Bellaloui et al., 2011). A two-year study in Stoneville, Mississippi, noted that applying nitrogen at a rate of 135 kg ha_-1_ significantly increased the linoleic acid levels compared to unfertilized plots in silt loam soil in 2015 (Kaur et al., 2017). However, the concentration of linolenic acids decreased in clay soil in the same study. Other studies have also documented a minor or no effect on soybean fatty acid composition with the application of nitrogen (Rahim et al., 2015; Szostak et al., 2020; Tamagno et al., 2020). All of these studies suggest that nitrogen application plays a role in determining the composition of soybean seeds. Due to the limited studies on soybean seed composition, particularly regarding the combined effects of biochar and nitrogen, this study aims to incorporate both factors to examine their impact on soybean seed composition, with a focus on the concentrations of fatty acids and amino acids in soybean seeds.

## 2 Materials and methods

### 2.1 Site description

A two-year field experiment was conducted during the 2023 and 2024 growing seasons (May-October) at the Kentucky State University Harold R. Benson Research and Demonstration Farm, located in Frankfort, Kentucky, USA (38° 07.153’ N, 84° 53.360’ W; elevation 250 m above sea level). The soil at the experimental site was classified as silt loam, comprising 9.91% sand, 71.47% silt, and 18.63% clay, with no prior history of soybean cultivation. The soil had a pH of 5.95, a total nitrogen content of 0.189%, a total carbon of 1.74%, and a cation exchange capacity (CEC) of 16.08 meq 100 g^-1^. Available phosphorus and potassium levels were 266.20 and 133.66 kg ha^-1^, respectively, which were measured using the Mehlich III extraction method (Mehlich, 1984). Field capacity and wilting point water contents were 39.27% and 19.35%, respectively, resulting in a plant-available water content of 19.92%.

### 2.2 Experimental design and treatments

The experiment was conducted using a split-split plot randomized complete block design (RCBD) with four replicates each year. Biochar application was the main plot factor. Cultivar served as the subplot factor, and nitrogen fertilization was the sub-subplot factor. Biochar and cultivar treatment assignments were maintained in the same plots in both years, as the biochar incorporated into the soil remained in place for the second season. Nitrogen treatments were re-randomized within each cultivar subplot in 2024 to minimize any potential positional effects from the previous year’s application. Each replication included 16 experimental plots (2 soil amendment levels × 2 cultivars × 4 nitrogen rates, randomly assigned), totaling 64 experimental units. The individual plots measured 7.3 m (24 ft) in length and were separated by 0.91 m (3 ft) alleys to prevent interference during management and harvesting.

Biochar was applied as the main plot factor at two levels: 0 t ha^-1^ (control, no biochar) and 12 t ha^-1^. Two of the four blocks received biochar amendment, while the remaining two served as non-amended controls. The biochar was surface-applied and subsequently incorporated into the topsoil about one month before planting in both years, providing enough time for initial soil equilibration. The biochar used in this study was produced from southern yellow pine (*Pinus* spp.) sawdust via pyrolysis at 650 °C for 10 minutes. The detailed physicochemical properties of the biochar, measured under dry conditions, are summarized in **Table 1**. Two soybean cultivars representing different maturity groups were selected as the subplot factor: a short-season cultivar from Maturity Group 2 (MG2) and a long-season cultivar from Maturity Group 4 (MG4). These cultivars were chosen based on their regional suitability to central Kentucky, yield potential, and performance under various environmental stress conditions. Each plot consisted of five rows spaced 38 cm (15 inches) apart, with a target seeding rate of 35 seeds per square meter. In 2023, MG2 cultivar was planted on May 14, and MG4 cultivar was planted one week later, on May 21. In 2024, both cultivars were planted simultaneously on May 21. The crop was grown entirely under rainfed conditions without supplemental irrigation in both growing seasons.

**Table 1:** Physical and chemical properties of southern yellow pine sawdust biochar (dry basis) used in the study.

Biochar property	Value (dry basis)
pH (25% mixture)	9.88
Fixed carbon (%)	90.58
Ash content (%)	3.31
Volatile matter (%)	6.11
Carbon content (%)	90.90
Nitrogen content (%)	0.40
Hydrogen content (%)	0.63
Sulfur content (%)	0.006
Oxygen content (%)	4.76
H:C (org) ratio	0.08
Density, when packed (lbs ft^-3^)	27.4

Biochar was sourced from Wakefield Biochar (Columbia, MO) and analyzed by Hazen Research, Inc.

Nitrogen was applied as the sub-subplot factor using urea (46-0-0) as the nitrogen source. Four nitrogen application rates were tested: 0 (unfertilized control), 40, 80, and 120 kg N ha^-1^. Nitrogen was intentionally applied late in the season, starting at the R5 (beginning seed) growth stage as defined by Fehr et al. (1971). The R5 stage marks the onset of rapid seed filling, when nitrogen demand is very high to support protein synthesis in developing seeds. At this stage, the plant redistributes nitrogen from vegetative tissues to meet nearly half of the seed’s nutritional needs, with the rest supplied by continued nitrogen fixation and root uptake (Gaspar et al., 2017). The reason for late-season application was based on growing evidence that biological nitrogen fixation alone may not meet the nitrogen requirements of modern high-yielding soybean cultivars during seed filling (Salvagiotti et al., 2008; Cafaro La Menza et al., 2017; Chiluwal et al., 2021). Each total nitrogen dose was split into three equal applications to maintain nitrogen availability during seed filling and to reduce the risk of nitrogen loss through volatilization or leaching: one at the R5 stage, a second one week after R5, and a third two weeks after R5. Fertilizer was carefully measured for each plot and applied manually between rows to ensure even distribution across the sub-subplot area.

### 2.3 Soil sampling and crop management

Before planting each year, each of the four blocks was divided into four subsections, and within each subsection, six soil cores were collected at a depth of 0–15 cm using a tube auger and composited into one representative sample, yielding 16 composite soil samples in total (4 blocks × 4 subsections). Analyses were performed at the University of Kentucky Regulatory Services Soil Testing Laboratory. Based on the soil test results, no phosphorus fertilizer was applied as soil phosphorus levels were already adequate (266.20 kg ha^-1^). Potassium was applied as muriate of potash (0-0-60) uniformly to all plots in both years to prevent these nutrients from limiting growth, thus isolating the effects of the primary experimental treatments (biochar, cultivar, and nitrogen) on seed composition. Weed management during the soybean growing season consisted of a single foliar application of a 2.5% glyphosate solution applied aerially via drone in both seasons. After the 2023 harvest, crop residues were incorporated into the soil, and the experimental plots were left fallow through the off-season before the 2024 planting. No additional pest or disease management interventions were necessary during either growing season.

### 2.4 Harvesting and seed composition analysis

A standardized harvest area was established within each plot to minimize border effects and ensure representative sampling. A length of 3.05 m (10 ft) was measured from the center of each plot, and the three central rows were selected for harvest, creating an effective harvest area of approximately 3.48 m² per plot. All plants outside the designated harvest zone were removed manually the day prior to mechanical harvesting to prevent seed contamination during combine operation. In 2023, MG2 cultivar was harvested in mid-September, and MG4 cultivar was harvested approximately one week later. In 2024, MG2 cultivar was harvested on September 28, and MG4 cultivar was harvested on October 5. After harvesting, seed samples from each experimental unit were brought to the Laboratory for compositional analysis.

The fatty acid and amino acid compositions of soybean seeds from each of the 64 experimental plots were analyzed using a DA 7250™ near-infrared (NIR) third-generation diode array analyzer (PerkinElmer/Perten Instruments, Springfield, IL, USA). The DA 7250™ is a fast, non-destructive device designed for at-line and laboratory testing in the food and agriculture sectors. It features a silicon and indium gallium arsenide (InGaAs) dual-diode array detector that operates in the 950–1650 nm wavelength range and performs multicomponent analysis in about 6 seconds per sample without requiring grinding or other sample preparation (PerkinElmer). The instrument is equipped with factory-developed calibration models based on a global database of hundreds of thousands of samples, and has been specifically validated for measuring moisture, protein, oil, fatty acid profiles, and amino acid levels in whole soybeans and other oilseeds (Bellaloui et al., 2011; Chiluwal et al., 2021). For each sample, seeds were placed directly into the large rotating tray of the DA 7250™ without grinding, and analysis was automatically initiated once the tray was in position. Each sample was scanned five times to ensure measurement consistency, and the average values were recorded. Seed samples were analyzed based on dry weight. The concentrations of individual fatty acids [palmitic acid (C16:0), stearic acid (C18:0), oleic acid (C18:1), linoleic acid (C18:2), and linolenic acid (C18:3)] were expressed in percentage of total fatty acids (oil), and individual amino acids were expressed as percentages of total seed dry weight.

### 2.5 Statistical analysis

All the concentrations of fatty acids and amino acids were analyzed for significance using linear mixed models in R (version 4.5.3) software built under the lme4 package (Bates et al., 2015). Year, biochar application, nitrogen application, cultivar MG nested within the year, and cultivar nested within MG and year, and their interactions were considered as fixed factors in the model. Replication (block), which was nested within the year, and plot, which was nested within replication, and their interactions with other fixed effects were considered as random factors. The means were calculated using the emmeans function, and Tukey at the 5% level of significance was used for mean separation. To highlight significant differences among treatments, various lower-case letters were used. Pearson’s correlation coefficients were computed among all amino acid and fatty acid concentrations using the cor() function in R to evaluate the strength and direction of linear relationships among seed composition traits. Correlations were tested for significance at p < 0.05, p < 0.01, and p < 0.001 levels using the cor.test() function, and the results were visualized as a lower-triangle correlation heatmap using the corrplot package.

## 3 Results

### 3.1 Climatic conditions

**Figure 1:**
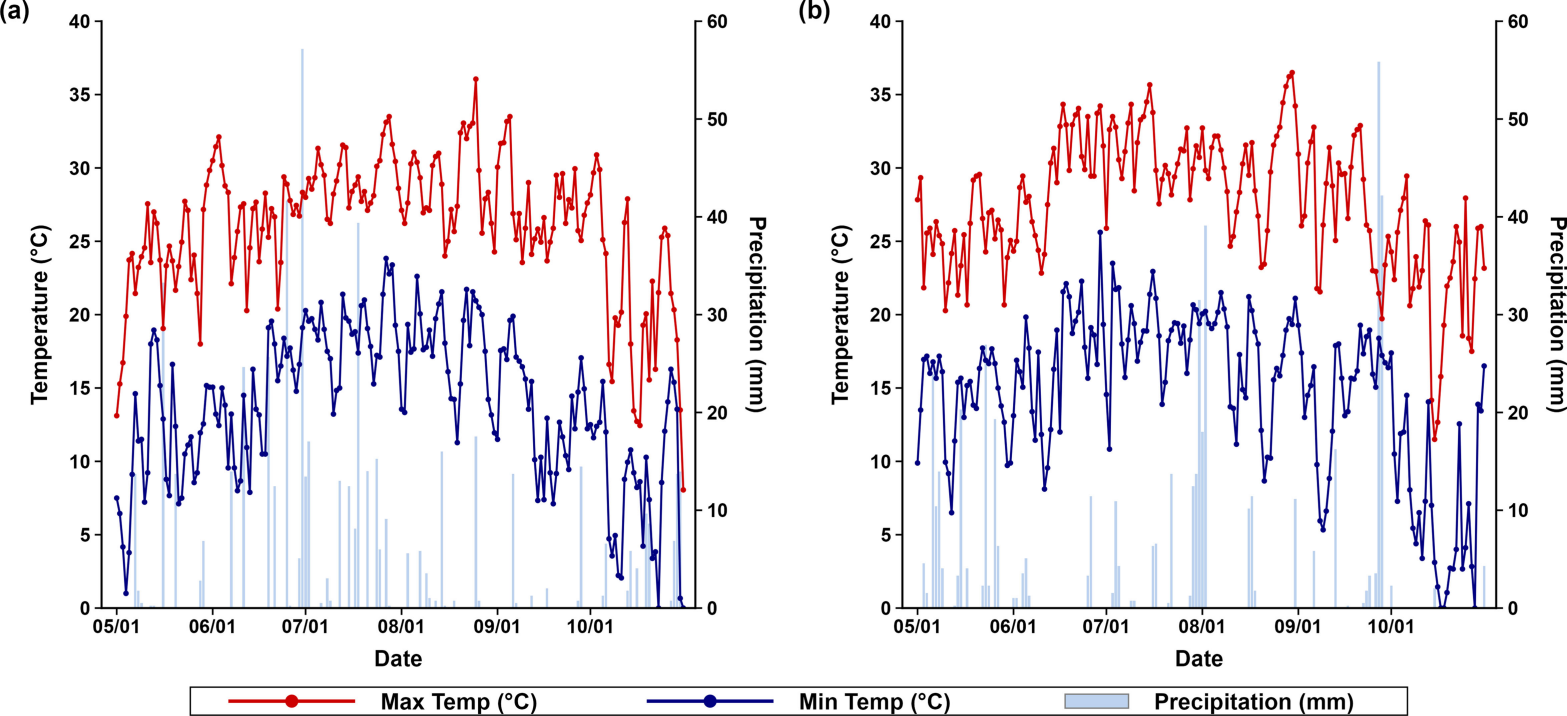
Daily maximum temperature (red line), minimum temperature (blue line), and precipitation (bars) during the soybean growing season (May-October) in **(a)** 2023 and **(b)** 2024 at the Harold R. Benson Research and Demonstration Farm, Kentucky State University, Frankfort, KY (38°07.153′N, 84°53.360′W, 250 m asl).

The experimental site is located within the humid subtropical climate zone, characterized by warm, humid summers and consistent year-round rainfall. During the 2023 growing season (May-October), about 49.86% of the total annual rainfall occurred within this period, with an average air temperature of 19.96 °C and a total rainfall of 522.2 mm (**Figure 1a**). Maximum temperatures in 2023 often surpassed 30 °C from June through September, while minimum temperatures generally ranged between 10 and 20 °C. An intense rainfall event took place in early July 2023, with a single-day rainfall exceeding 55 mm, followed by a drier period through August (**Figure 1a**). In 2024, the same period accounted for 42.42% of the year’s total rainfall, with a slightly higher average temperature of 21.04 °C and a lower total rainfall of 495.8 mm (**Figure 1b**). The 2024 season experienced more sustained high maximum temperatures from late June through early September, with peaks nearing 35 °C (**Figure 1b**). Rainfall in 2024 appeared more concentrated toward the late season (September-October) compared to 2023, which received more consistent mid-season rainfall.

### 3.2 Essential amino acid composition

The analysis of variance showed that late-season nitrogen fertilization was the most effective treatment factor on soybean amino acid composition, depending on the year and cultivar (**Table 2**). It significantly affected seven out of ten essential amino acids analyzed in this study (**Table 3**). Cultivar performance also varied by year for seven amino acids (**Table 2**). Biochar amendment at 12 t ha^-1^ did not significantly influence any amino acid (**Table 2**). The value range for observed amino acid concentrations per seed dry weight were: methionine (0.50-0.54%), cysteine (0.50-0.57%), threonine (1.44-1.55%), lysine (2.38-2.67%), valine (1.90-2.07%), leucine (2.95-3.22%), isoleucine (1.85-2.02%), phenylalanine (2.00-2.20%), histidine (1.00-1.09%), and tryptophan (0.39-0.45%).

**Table 2:** Analysis of variance (p-values) for amino acid concentrations as affected by year (Y), biochar (B), cultivar (C), and nitrogen (N) treatments.

Source of variation	Methionine	Cysteine	Threonine	Lysine	Tryptophan	Valine	Leucine	Isoleucine	Phenylalanine	Histidine
Year (Y)	0.343	0.525	0.223	0.035*	0.627	0.547	0.333	0.419	0.685	0.824
Biochar (B)	0.432	0.099	0.937	0.820	0.786	0.768	0.969	0.706	0.867	0.831
Cultivar (C)	0.029*	0.086	0.237	0.227	0.183	0.103	0.093	0.078	0.355	0.307
Nitrogen (N)	0.386	0.358	<0.001***	0.003**	0.162	<0.001***	<0.001***	0.006**	<0.001***	<0.001***
Y × B	0.872	0.562	0.381	0.428	0.554	0.588	0.761	0.989	0.738	0.953
Y × C	0.143	0.703	0.002**	0.002**	0.342	<0.001***	<0.001***	<0.001***	<0.001***	0.003**
Y × N	0.096	0.027*	0.002**	0.017*	0.394	0.028*	0.005**	0.091	0.020*	<0.001***
B × C	0.051	0.115	0.560	0.398	0.131	0.212	0.584	0.205	0.738	0.358
B × N	0.724	0.530	0.674	0.496	0.878	0.882	0.850	0.954	0.973	0.593
C × N	0.945	0.930	0.214	0.091	0.925	0.096	0.049*	0.007**	0.108	0.181
Y × B × C	0.809	0.779	0.695	0.865	0.131	0.790	0.833	0.812	0.756	0.892
Y × B × N	0.740	0.798	0.561	0.769	0.478	0.335	0.628	0.656	0.378	0.711
Y × C × N	0.327	0.703	0.926	0.280	0.784	0.221	0.350	0.589	0.783	0.693
B × C × N	0.474	0.711	0.268	0.188	0.884	0.661	0.534	0.926	0.713	0.851
Y × B × C × N	0.744	0.398	0.329	0.347	0.372	0.901	0.527	0.720	0.437	0.596

*, **, *** indicate significance at p < 0.05, p < 0.01, and p < 0.001, respectively.

**Table 3:** Amino acid concentrations (% seed dry weight) as affected by nitrogen (N), year × nitrogen (Y × N), cultivar × nitrogen (C × N), and year × cultivar (Y × C).

Nitrogen (N)										
Treatment	Met	Cys	Thr	Lys	Trp	Val	Leu	Ile	Phe	His
N0	0.519	0.531	1.489b	2.527b	0.425	1.973b	3.066b	1.935b	2.085b	1.038b
N40	0.522	0.535	1.496ab	2.533ab	0.427	1.983ab	3.079b	1.945ab	2.096b	1.042b
N80	0.525	0.535	1.511a	2.556a	0.421	2.003a	3.118a	1.959a	2.122a	1.060a
N120	0.522	0.526	1.509a	2.555a	0.412	2.003a	3.114a	1.960a	2.122a	1.059a

Year × Nitrogen (Y × N)										
Treatment	Met	Cys	Thr	Lys	Trp	Val	Leu	Ile	Phe	His
2023 × N0	0.523	0.526ab	1.503ab	2.596ab	0.426	1.976ab	3.082ab	1.944	2.089ab	1.041ab
2023 × N40	0.532	0.539a	1.520ab	2.624ab	0.428	2.004ab	3.120ab	1.966	2.116ab	1.058ab
2023 × N80	0.530	0.531ab	1.518ab	2.619ab	0.422	2.007ab	3.122ab	1.962	2.120ab	1.054ab
2023 × N120	0.524	0.514b	1.511ab	2.622ab	0.402	2.002ab	3.120ab	1.963	2.119ab	1.055ab
2024 × N0	0.514	0.536ab	1.476b	2.458ab	0.423	1.971ab	3.049b	1.926	2.081b	1.035b
2024 × N40	0.511	0.530ab	1.472b	2.442b	0.426	1.961b	3.039b	1.924	2.076b	1.026b
2024 × N80	0.519	0.539ab	1.504a	2.494a	0.421	1.999a	3.114a	1.957	2.125a	1.067a
2024 × N120	0.520	0.539ab	1.507a	2.489a	0.421	2.004a	3.109a	1.958	2.126a	1.062a

Cultivar × Nitrogen (C × N)										
Treatment	Met	Cys	Thr	Lys	Trp	Val	Leu	Ile	Phe	His
MG2 × N0	0.524	0.534	1.499	2.552	0.429	1.992	3.100abc	1.961a	2.101	1.049
MG2 × N40	0.526	0.541	1.498	2.536	0.429	1.982	3.089bc	1.951ab	2.094	1.044
MG2 × N80	0.531	0.542	1.511	2.561	0.424	2.011	3.125ab	1.958ab	2.123	1.061
MG2 × N120	0.528	0.533	1.518	2.565	0.418	2.018	3.141a	1.976a	2.133	1.063
MG4 × N0	0.513	0.527	1.480	2.502	0.420	1.954	3.032c	1.909b	2.069	1.028
MG4 × N40	0.518	0.528	1.494	2.530	0.425	1.983	3.070abc	1.939ab	2.098	1.040
MG4 × N80	0.519	0.528	1.511	2.551	0.418	1.995	3.111ab	1.961a	2.122	1.060
MG4 × N120	0.516	0.519	1.500	2.546	0.405	1.989	3.088ab	1.945ab	2.112	1.054

Year × Cultivar (Y × C)										
Treatment	Met	Cys	Thr	Lys	Trp	Val	Leu	Ile	Phe	His
2023 × MG2	0.529	0.532	1.498ab	2.585a	0.421	1.965b d	3.061ab	1.928ab	2.066bc	1.038ab
2023 × MG4	0.525	0.522	1.528ab	2.645a	0.418	2.029a c	3.161a	1.989a	2.156a	1.066ab
2024 × MG2	0.525	0.543	1.515a	2.522a	0.429	2.036ab	3.166a	1.994a	2.159ab	1.070a
2024 × MG4	0.508	0.529	1.465b	2.419b	0.416	1.931cd	2.989b	1.888b	2.044c	1.025b

Means followed by different letters within a column and section differ significantly (Tukey HSD, p < 0.05). Values without letters indicate non-significant effects. Met, methionine; Cys, cysteine; Thr, threonine; Lys, lysine; Trp, tryptophan; Val, valine; Leu, leucine; Ile, isoleucine; Phe, phenylalanine; His, histidine.

**Figure 2:**
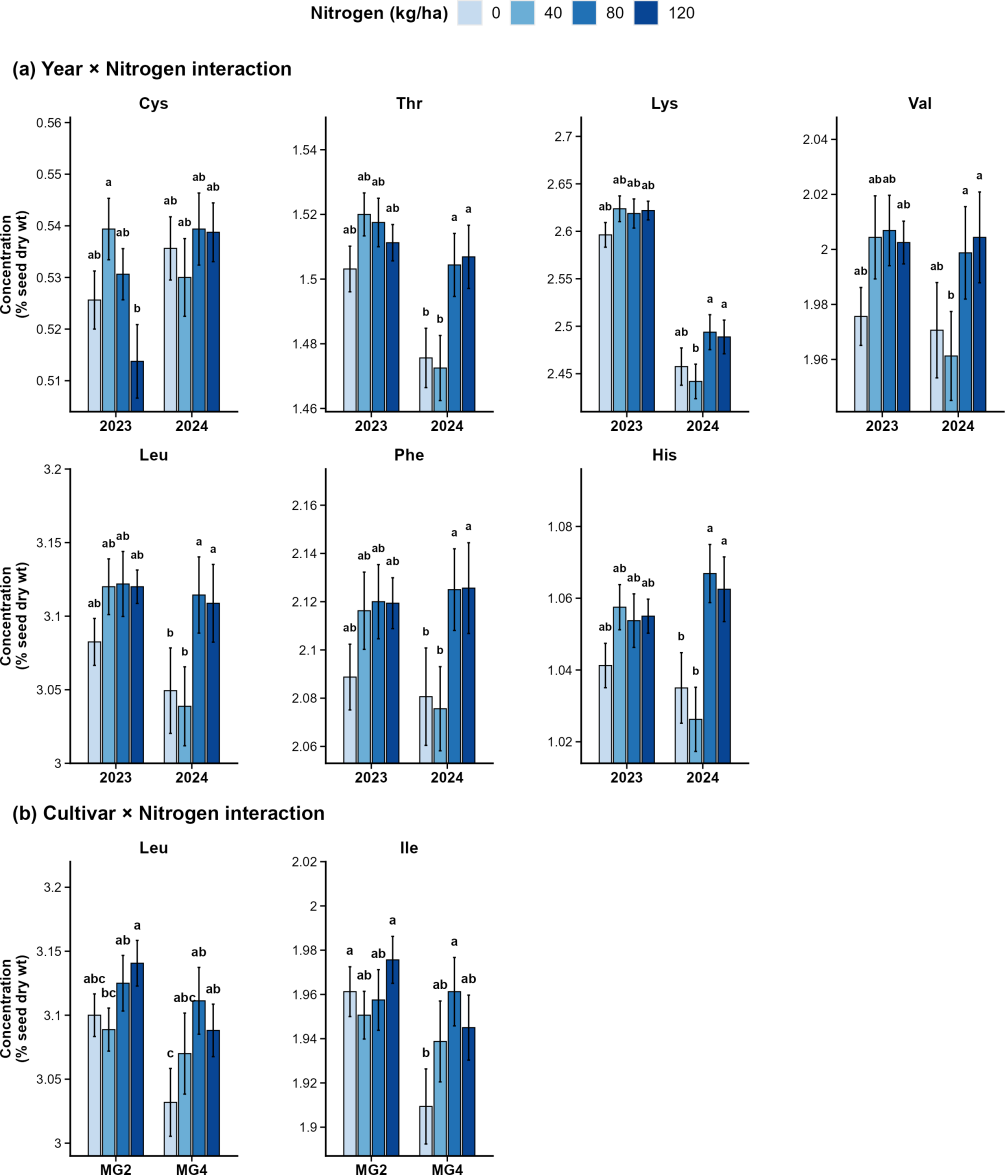
Effect of year × nitrogen **(a)** and cultivar × nitrogen **(b)** interactions on amino acid concentrations (% seed dry weight) in soybean seeds. Bars represent means and error bars indicate ± SE. Different letters above bars within each panel indicate significant differences at p < 0.05. Cys, cysteine; Thr, threonine; Lys, lysine; Val, valine; Leu, leucine; Phe, phenylalanine; His, histidine; Ile, isoleucine.

Nitrogen application significantly increased the concentrations of threonine (p < 0.001), valine (p < 0.001), leucine (p < 0.001), phenylalanine (p < 0.001), histidine (p < 0.001), lysine (p = 0.003), and isoleucine (p = 0.006) (**Table 3**). Across all seven responsive amino acids, concentrations increased from N0 to N80 and then plateaued at N120 with no further gain observed. This indicates that 80 kg N ha^-1^ represents an effective threshold for improving these amino acid concentrations. The sulfur-containing amino acids methionine (p = 0.386) and cysteine (p = 0.358) were not significantly affected by the main effect of nitrogen application (**Table 2**). Methionine ranged narrowly from 0.519% at N0 to 0.525% at N80, while cysteine showed no consistent dose-response (**Table 3**). The nitrogen effect on amino acids was not consistent across years, as indicated by significant year × nitrogen interactions for cysteine (p = 0.027), threonine (p = 0.002), lysine (p = 0.017), valine (p = 0.028), leucine (p = 0.005), phenylalanine (p = 0.020), and histidine (p < 0.001) (**Table 3**; **Figure 2a**). For cysteine, the year × nitrogen interaction followed a different trend than the others: in 2023, the highest concentration was at N40 (0.539 ± 0.0059%) than at N120 (0.514 ± 0.0071%), whereas in 2024, no significant differences were observed among nitrogen rates (**Table 3**). For other amino acids in 2023, the values were statistically similar to each other, with no difference observed with or without fertilization, whereas in 2024, the values were higher in N80 and N120 than in N40 and no fertilization. In 2024, nitrogen application at N80 and N120 increased essential amino acids (excluding cysteine) by approximately 1.3% to 3.1% relative to N0, with the highest improvement observed for histidine (**Figure 2a**). Nitrogen application also significantly affected leucine (p = 0.049) and isoleucine (p = 0.007) across cultivars (**Table 3**; **Figure 2b**). In the MG2 cultivar, leucine at N120 increased by approximately 1.7% compared to N40, while in the MG4 cultivar, leucine at N80 and N120 was about 2.6% and 1.8% higher, respectively, than N0. Similarly, isoleucine concentration increased with nitrogen application, with N80 showing an increase of approximately 2.7% over N0 in 2024, whereas no significant differences were observed among treatments in 2023.

Cultivar performance also varied by year for threonine (p = 0.002), lysine (p = 0.002), valine (p < 0.001), leucine (p < 0.001), isoleucine (p < 0.001), phenylalanine (p < 0.001), and histidine (p = 0.003) (**Table 3**). In 2023, concentrations of most amino acids were similar between cultivars, except for valine and phenylalanine. However, in 2024, the MG2 cultivar produced significantly higher concentrations than the MG4 cultivar for all seven amino acids. Relative to MG4, MG2 cultivar concentrations in 2024 were higher by 3.4% for threonine, 4.3% for lysine, 5.4% for valine, 5.9% for leucine, 5.6% for isoleucine, 5.6% for phenylalanine, and 4.4% for histidine (**Table 3**). Biochar application at 12 t ha^-1^ did not significantly affect any of the ten amino acids in this study (**Table 2**). All interactions involving biochar were also not significant.

### 3.3 Fatty acid composition

**Figure 3:**
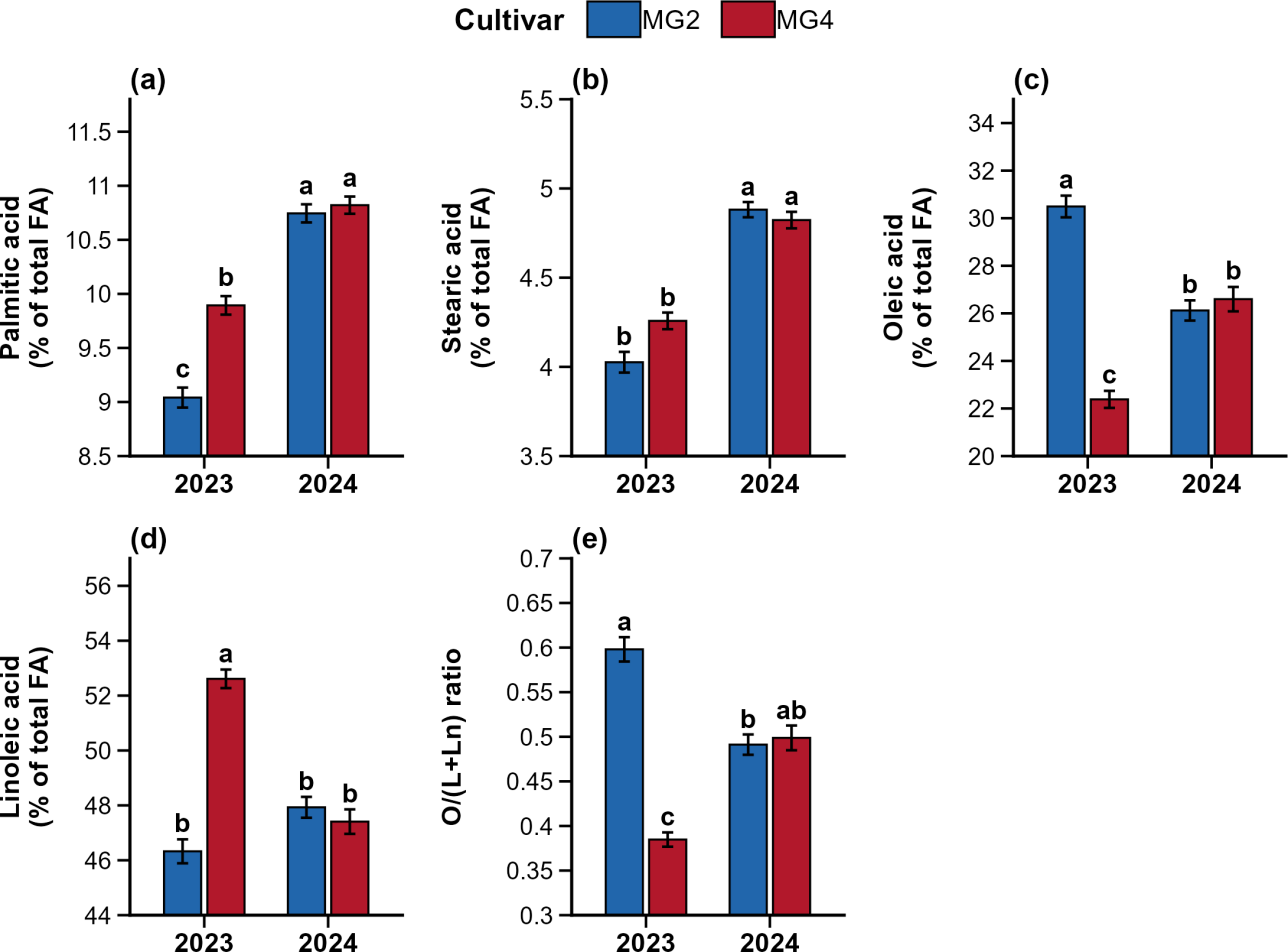
Effect of year × cultivar interaction on fatty acid concentrations (% of total fatty acids) and oleic to polyunsaturated fatty acid ratio [O/(L+Ln)] in soybean seeds during 2023 and 2024. **(a)** Palmitic acid, **(b)** stearic acid, **(c)** oleic acid, **(d)** linoleic acid, and **(e)** O/(L+Ln) ratio. Bars represent means of MG2 (blue) and MG4 (red) cultivars, and error bars indicate ± SE. Different letters above bars indicate significant differences at p < 0.05.

The ANOVA showed main effects of year, cultivar, and nitrogen on fatty acid concentration (**Table 4**). Compared to 2023, 2024 showed a significant relative increase (p ≤ 0.014) in palmitic acid (13.8%) and stearic acid (17.1%). The main effect of nitrogen was only found in oleic acid (p = 0.048) with no other interaction. Nitrogen application at 80 kg ha^-1^ (27.42 ± 0.59) increased oleic acid concentration by 4.9% relative to N0 (26.13 ± 0.66) and by 6.3% relative to N40 (25.79 ± 0.70) (**Table 5**). Cultivar performance over two year was the most influential factor governing fatty acid composition, significantly affecting palmitic (p < 0.001), stearic (p = 0.002), oleic (p = 0.001), linoleic (p = 0.003), and the oleic to polyunsaturated fatty acid ratio [O/(L+Ln)] (p = 0.002) (**Figure 3**). Biochar amendment at 12 t ha^-1^ did not significantly affect any fatty acid or the O/(L+Ln) ratio either as main or in interaction (**Table 4**).

**Table 4:** Analysis of variance (p-values) for fatty acid concentrations (% of total fatty acids) and oleic to polyunsaturated fatty acid ratio [O/(L+Ln)] as affected by year (Y), biochar (B), cultivar (C), and nitrogen (N) treatments.

Source of Variation	Palmitic	Stearic	Oleic	Linoleic	Linolenic	O/(L+Ln)
Year (Y)	0.014*	0.013*	0.908	0.161	0.085	0.869
Biochar (B)	0.973	0.572	0.261	0.438	0.856	0.290
Cultivar (C)	<0.001***	0.060	0.002**	0.005**	<0.001***	0.003**
Nitrogen (N)	0.120	0.112	0.048*	0.553	0.064	0.110
Y × B	0.267	0.297	0.941	0.873	0.315	0.995
Y × C	<0.001***	0.002**	0.001**	0.003**	0.985	0.002**
Y × N	0.802	0.283	0.284	0.812	0.229	0.354
B × C	0.609	0.992	0.318	0.620	0.285	0.349
B × N	0.201	0.934	0.476	0.809	0.717	0.632
C × N	0.066	0.072	0.770	0.863	0.365	0.840
Y × B × C	0.327	0.725	0.546	0.909	0.356	0.580
Y × B × N	0.465	0.281	0.471	0.161	0.724	0.377
Y × C × N	0.115	0.524	0.231	0.059	0.085	0.196
B × C × N	0.252	0.258	0.732	0.870	0.553	0.865
Y × B × C × N	0.563	0.067	0.907	0.428	0.280	0.791

*, **, *** indicate significance at p < 0.05, p < 0.01, and p < 0.001, respectively. O/(L+Ln), ratio of oleic acid to the sum of linoleic and linolenic acids.

**Table 5:** Fatty acid concentrations (% of total fatty acids) and oleic to polyunsaturated fatty acid ratio [O/(L+Ln)] as affected by biochar (B), nitrogen (N), cultivar (C), and year × cultivar (Y × C).

Biochar (B)						
Treatment	Palmitic	Stearic	Oleic	Linoleic	Linolenic	O/(L+Ln)
Biochar	10.12	4.52	26.84	48.22	5.61	0.506
Control	10.13	4.48	25.95	48.92	5.66	0.481

Nitrogen (N)						
Treatment	Palmitic	Stearic	Oleic	Linoleic	Linolenic	O/(L+Ln)
N0	10.07	4.53	26.13b	48.62	5.87	0.485
N40	10.28	4.55	25.79b	48.69	5.69	0.480
N80	10.13	4.50	27.42a	48.11	5.45	0.517
N120	10.02	4.40	26.25ab	48.86	5.54	0.490

Cultivar (C)						
Treatment	Palmitic	Stearic	Oleic	Linoleic	Linolenic	O/(L+Ln)
MG2	9.89b	4.45	28.31a	47.13b	5.24b	0.545a
MG4	10.36a	4.54	24.49b	50.01a	6.03a	0.442b

Year × Cultivar (Y × C)						
Treatment	Palmitic	Stearic	Oleic	Linoleic	Linolenic	O/(L+Ln)
2023 × MG2	9.04c	4.03b	30.49a	46.33b	4.96	0.598a
2023 × MG4	9.89b	4.26b	22.38c	52.61a	5.75	0.385c
2024 × MG2	10.74a	4.88a	26.12b	47.93b	5.52	0.491b
2024 × MG4	10.82a	4.82a	26.60b	47.41b	6.31	0.499ab

Means followed by different letters within a column and section differ significantly. Values without letters indicate non-significant effects. O/(L+Ln), ratio of oleic acid to the sum of linoleic and linolenic acids. Fatty acid concentrations are expressed as a percentage of total fatty acids.

The year × cultivar interaction for palmitic, stearic, oleic, linoleic, and O/(L+Ln) showed significant cultivar responses only in the 2023 growing season (**Figure 3**). This year, a significantly higher value was found only for oleic acid in the MG2 cultivar, which was 36.2% higher than in MG4. But the MG4 cultivar showed higher concentrations of palmitic and linoleic acids by about 9.4% and 13.6%, respectively, compared to MG2. The high oleic acid value resulted O/(L+Ln) ratio 55.3% higher in the MG2 cultivar than MG4 in 2023. Linolenic acid was the only fatty acid for which cultivar showed a significant main effect (p < 0.001) without a year × cultivar interaction (p = 0.985) with a 15.1% higher value in the MG2 cultivar (**Table 5**).

### 3.4 Correlation among amino acids and fatty acids

**Figure 4:**
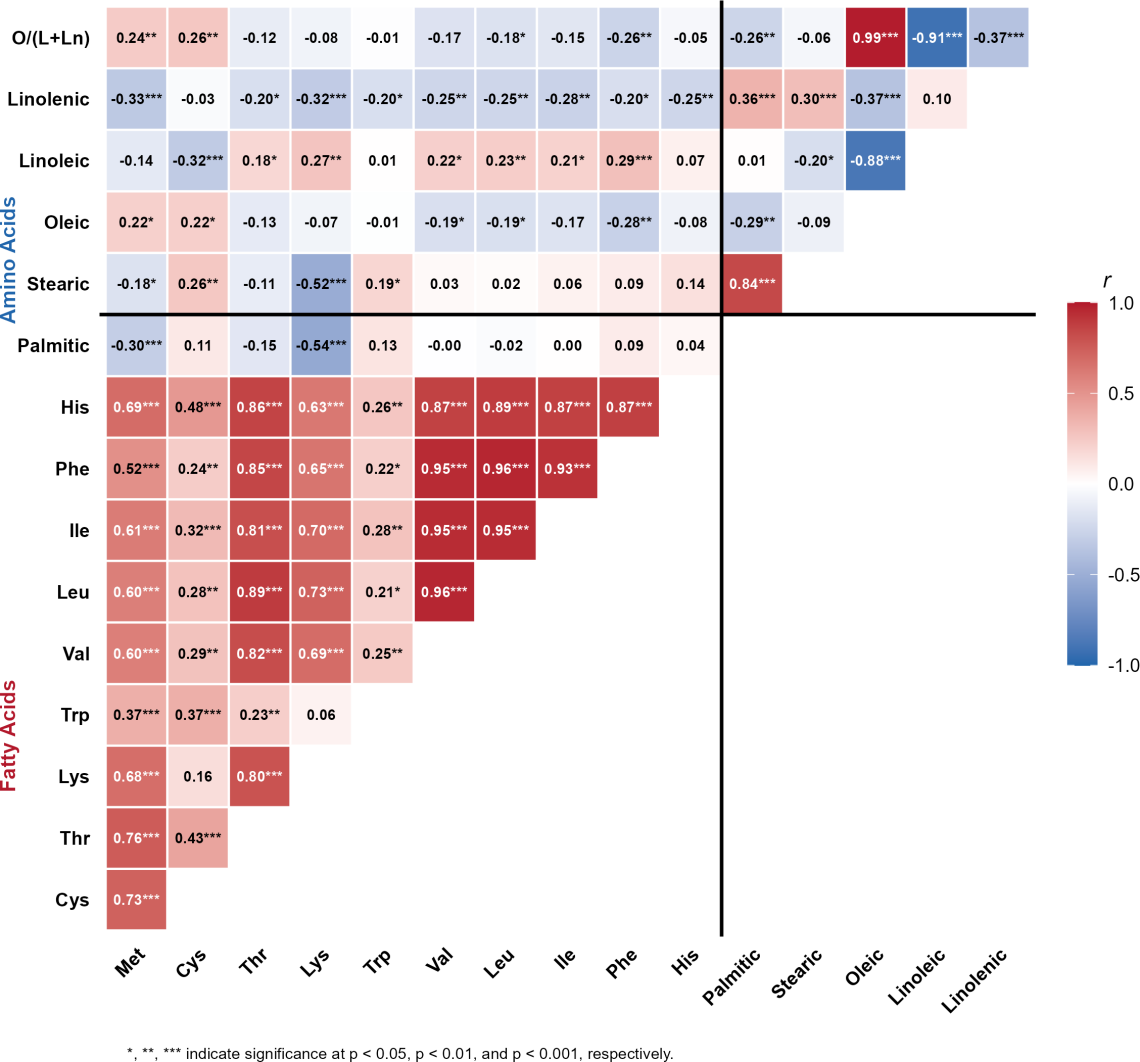
Pearson correlation matrix among amino acid concentrations (% seed dry weight) and fatty acid concentrations (% of total fatty acids). Met, methionine; Cys, cysteine; Thr, threonine; Lys, lysine; Trp, tryptophan; Val, valine; Leu, leucine; Ile, isoleucine; Phe, phenylalanine; His, histidine; O/(L+Ln), ratio of oleic acid to the sum of linoleic and linolenic acids. *, **, *** indicate significance at p < 0.05, p < 0.01, and p < 0.001, respectively.

Pearson correlation analysis showed distinct patterns of association among the seed composition traits (**Figure 4**). Among the amino acids, the seven nitrogen-responsive ones (Thr, Lys, Val, Leu, Ile, Phe, and His) were strongly and positively correlated with each other (r = 0.81-0.96, p < 0.001). The strongest pairwise correlations were observed between leucine and valine (r = 0.96, p < 0.001), leucine and isoleucine (r = 0.95, p < 0.001), and isoleucine and phenylalanine (r = 0.95, p < 0.001). Methionine was strongly correlated with cysteine (r = 0.73, p < 0.001) and threonine (r = 0.76, p < 0.001) but showed weaker associations with the branched-chain amino acids. Tryptophan showed the weakest correlations with other amino acids (r = 0.06-0.37), consistent with its non-significant response to nitrogen fertilization. Among the fatty acids, an inverse relationship between oleic and linoleic acid was observed (r = −0.88, p < 0.001). Palmitic and stearic acids were highly positively correlated (r = 0.84, p < 0.001). The O/(L+Ln) ratio was almost entirely determined by oleic acid (r = 0.99, p < 0.001) and was strongly negatively correlated with linoleic acid (r = −0.91, p < 0.001). Correlations between amino acid and fatty acid groups were generally weak. Methionine showed modest positive correlations with oleic acid (r = 0.22, p < 0.05) and negative correlations with palmitic acid (r = −0.30, p < 0.001) and linolenic acid (r = −0.33, p < 0.001). Most other amino acid-fatty acid correlations were either nonsignificant or below r = 0.30.

## 4 Discussion

The lack of a significant response or even a reduction at the higher dose (N120) in one of the study years for the sulfur-containing amino acids, methionine and cysteine, in relation to nitrogen application, is a key observation from a feed quality perspective because methionine and cysteine are the most limiting amino acids in soybean meal for poultry and swine (Medic et al., 2014; Warrington et al., 2015). A study has provided a mechanistic explanation for the reduction of sulfur-containing amino acids using fluorescence two-dimensional difference gel electrophoresis (Krishnan et al., 2005). They found that nitrogen application favored the formation of the β-subunit of β-conglycinin (a sulfur-poor storage protein), whereas it decreased the accumulation of Bowman-Birk protease inhibitor (BBI), a cysteine-rich protein containing 14 cysteine residues per molecule. They have also found that nitrogen application increased protein content but reduced levels of sulfur-containing amino acids. Pfarr et al. (2018) further supported this phenomenon where they found the negative relationship between total protein content and sulfur amino acid concentration. Therefore, sulfur fertilization might be the appropriate strategy for improving the most limiting sulfur-containing amino acids. Rushovich and Weil (2021) demonstrated that field-scale sulfur application significantly enhanced methionine and cysteine concentrations in soybean seeds, particularly in soils where sulfur deficiency was increasingly common due to reduced atmospheric deposition and depletion by high yields. A comprehensive meta-analysis across 44 site-years and 18 locations in eight US states found that sulfur application increased sulfur amino acid concentrations by approximately 1% (Borja Reis et al., 2021). More recently, Rahman et al. (2025) confirmed that sulfur and nitrogen fertilization can selectively enhance cysteine and methionine content. These findings suggest that integrating sulfur fertilization with late-season nitrogen application may be a promising strategy to increase the levels of sulfur-containing essential amino acids in soybean seeds. After methionine and cysteine, threonine and lysine are the limiting amino acids in soybean meal for monogastric animals (Farkhoy et al., 2012; Warrington et al., 2015). In the second growing season, our study found increased concentrations of threonine and lysine at N80 and N120, which suggests that late-season nitrogen fertilization could be a useful approach for quality management, depending upon the environmental conditions for certain essential amino acids. Climatic conditions during seed filling directly influenced amino acid responses in this study. Most amino acid concentrations have been shown to be positively correlated with mean daily temperature during seed filling (Song et al., 2016), although the relationship can be nonlinear and varies by individual amino acid (Carrera et al., 2011). The warmer and drier 2024 conditions also likely suppressed biological nitrogen fixation, increasing the crop’s reliance on supplemental nitrogen and explaining the stronger amino acid response to nitrogen that year.

For fatty acids, cultivar performance varied by the growing seasons. In 2023, the MG2 cultivar had 36.2% higher oleic acid and a 55.3% higher O/(L+Ln) ratio than MG4, but these differences between the cultivars were reduced in the warmer 2024 season. This phenomenon could be associated with the temperature sensitivity of the fatty acid desaturation pathway. Soybean fatty acid composition is mainly controlled by the activity of FAD2 desaturase, which converts oleic acid to linoleic acid, and FAD3 desaturase, which converts linoleic acid to linolenic acid (Reinprecht and Pauls, 2016; Dar et al., 2017). High temperature during seed filling reduces desaturase activity, resulting in increased oleic acid and decreased linoleic and linolenic acids (Cheesbrough, 1989; Dornbos and Mullen, 1992; Bellaloui et al., 2015). Bukowski and Goslee (2024) developed a temperature-response model using 233 data points from 16 studies and found that high-daytime temperatures during seed development reduce desaturase activity. The study observed decreased levels of linoleic and linolenic acids and increased levels of oleic acid. This temperature effect explains why MG4’s oleic acid increased sharply from 22.38% in 2023 to 26.60% in 2024, while linoleic acid declined from 52.61% to 47.41%. Nitrogen had a limited effect on fatty acids, significantly influencing only oleic acid (p = 0.048). Past studies have also reported that fatty acid composition is more influenced by genotype and environment rather than by nutrient management (Tamagno et al., 2020). The highly significant negative correlation between oleic and linoleic acids (r = -0.88; **Figure 4**) indicates their competitive relationship via the FAD2 desaturase pathway, where oleic acid acts as the substrate for linoleic acid synthesis.

Biochar application at 12 t ha^-1^ did not significantly affect any amino acid or fatty acid measured in this study (**Tables 2**, **4**). This differs from the controlled pot experiments that reported biochar alone significantly increased leucine and cysteine in soybean shoot tissues while decreasing methionine and isoleucine, whereas the combined application of biochar with the endophytic fungus significantly increased methionine, cysteine, isoleucine, and phenylalanine compared to the control (Waqas et al., 2017). The study also found that biochar alone increased palmitic, stearic, and oleic acids, while the biochar-endophyte combination increased linoleic and linolenic acids. The discrepancy might be due to differences in experimental scales and soil conditions. These results were obtained from soybean vegetative shoot tissues grown under controlled growth chamber conditions rather than from mature seeds under field conditions. Also, the experimental soil in our study had relatively adequate nutrient status (P = 266.20 kg ha^-1^, K = 133.66 kg ha^-1^, CEC = 16.08 meq 100 g^-1^, pH 5.95) and most documented biochar benefits on soil fertility and crop productivity have been reported in soils with inherent limitations, such as high acidity, sandy texture, alkalinity, low organic matter, or nutrient deficiency (Ma et al., 2019; Diatta et al., 2020; Xiu et al., 2021; Yin et al., 2021). Moreover, biochar may take time to incorporate into the soil as the material weathers and functional groups develop. Biochar effects on plant performance were mediated by improvements in water and nutrient use efficiency rather than by direct compositional changes. The two-year duration of the present study may have been insufficient for biochar to have measurable effects on seed composition. Long-term studies with various application rates or in combination with sulfur fertilization may reveal biochar’s potential to improve soybean nutritional quality.

## 5 Conclusions

This two-year field study showed that methionine, cysteine, and tryptophan were unresponsive to late-season nitrogen at R5, although 80 kg N ha^-1^ effectively improves threonine, lysine, valine, leucine, isoleucine, phenylalanine, and histidine. The results indicate that nitrogen alone cannot address the sulfur-containing amino acids deficiency, which remains the primary nutritional limitation of soybean meal. The nitrogen response was environment-dependent, with significant effects mainly in the warmer 2024 season, when biological nitrogen fixation was likely suppressed by drought during seed filling. Fatty acid composition was affected mainly by cultivar and year interaction. The MG2 cultivar had a higher O/(L+Ln) ratio than MG4, indicating that cultivar selection is important for improving oil oxidative stability. Nitrogen significantly influenced only oleic acid, with no significant effect on the O/(L+Ln) ratio, further supporting that the fat composition is more determined by genetics. Biochar at 12 t ha^-1^ did not affect any amino acid or fatty acid. Future research should focus on integrating sulfur fertilization with late-season nitrogen, while also evaluating longer-term impact of biochar across diverse soil types to enhance sulfur-containing amino acid concentrations and oil oxidative stability.

## Data Availability

The original contributions presented in the study are included in the article/supplementary material. Further inquiries can be directed to the corresponding author.
